# F87. SERUM PROLACTIN LEVELS AND COGNITIVE OUTCOME IN FIRST EPISODE PSYCHOSIS: A PROSPECTIVE 1-YEAR FOLLOW-UP STUDY

**DOI:** 10.1093/schbul/sby017.618

**Published:** 2018-04-01

**Authors:** Meritxell Tost Bonet, Itziar Montalvo, Francesc Estrada, Maria Isabel Ahuir Perez, Ángel Cabezas, Montse Paños, Montserrat Sole, José Antonio Monreal, vanessa Sanchez-gistau, Elisabet Vilella, Diego Palao, Javier Labad

**Affiliations:** 1 Parc Taulí Hospital Universitari, I3PT, Universitat Autònoma de Barcelona; 2 Early Intervention Service, Hospital Universitari Institut Pere Mata, IISPV, Universitat Rovira i Virgili

## Abstract

**Background:**

Recent studies in patients with psychotic disorders or prolactinomas suggest that increased prolactin levels may have negative effects on cognition. Most previous studies including patients with psychotic disorders are cross-sectional, and longitudinal studies are lacking. We aimed to conduct an observational, prospective study to explore whether prolactin levels during the first year of treatment are associated with changes in cognitive tasks. Our hypothesis would be that those patients with increased prolactin levels would show a poorer cognitive outcome than those patients with lower prolactin levels.

**Methods:**

We studied 60 patients (24.5 ± 6.8 years; 36% women) with a first episode psychosis (FEP) attending the Early Psychosis Programme from two institutions (Parc Taulí Hospital Universitari, Sabadell, Spain; Hospital Universitari Institut Pere Mata). Ethical approval was obtained from the local Ethics Committees of both institutions. Clinical diagnoses for a FEP were generated with the OPCRIT checklist v.4.0 after a semistructured interview by a psychiatrist. The MATRICS Consensus Cognitive Battery (MCCB) was administered to explore neuropsychological functioning at two-time points (baseline, 1 year). The MCCB contains 10 tests to measure cognitive performance in 7 cognitive domains. Three fasting blood samples (baseline, 6 months, 12 months) were obtained in the morning between 8:30 h and 9:30 h in resting conditions, to determine unstimulated plasma prolactin. Serum prolactin levels were determined with immunoassays standardized against the 3rd International Reference Standard 84/500. Statistical analyses were performed with SPSS version 21.0. As prolactin levels might be increased by stress, particularly at the onset of the FEP, we calculated mean prolactin levels over the follow-up period taking into account prolactin values at 6 and 12 months. A general linear model for repeated measures was performed in order to test whether longitudinal changes in cognitive tasks differed by mean prolactin levels. All analyses were adjusted for gender. A p value < 0.05 (two-tailed) was considered to be significant. For descriptive purposes, patients in the fourth quartile for serum prolactin (>47.8 ng/ml for men; >54.3 ng/ml for women) where compared with those with lower prolactin levels.

**Results:**

Patients improved in all 10 MCCB cognitive tasks one year later (p<0.05 for all tasks). When exploring the interaction between time x prolactin in the GLM analyses, significant interactions were found for three cognitive tasks related with processing speed (BACS-SC [F= 5.9, p= 0.018]; Fluency [F= 5.6, p= 0.022]) and reasoning and problem solving (NAB mazes [F= 4.8, p= 0.033]). Percent change over the follow-up period in these cognitive tasks was greater for patients with lower prolactin levels, when compared to those in the highest quartile: BACS-SC (11.8% vs 0.6%), Fluency (8.5% vs -7.4%) and NAB mazes (10.7% vs -1.1%).

**Discussion:**

Our study is in accordance with previous cross-sectional studies reporting a negative effect of prolactin levels on cognition and adds new information with a prospective design. A limitation of our study is that patients were treated with different antipsychotics based on the clinical routine practice. Antipsychotic-induced hyperprolactinaemia, which is caused by tuberoinfundibular blockade of D2 receptors, may be reflecting the blockade of D2 receptors in other dopaminergic pathways (striatum and mesocortical pathways) that may also affect cognitive abilities. Further clinical trials are needed to reduce the heterogeneity of the treatment effects and to confirm the potential negative effects of prolactin on cognitive abilities.

